# Endothelial PPARδ facilitates the post-ischemic vascular repair through interaction with HIF1α

**DOI:** 10.7150/thno.69017

**Published:** 2022-01-24

**Authors:** Yalan Wu, Xiaolong Tang, Sharen Lee, Huiling Hong, Xiaoyun Cao, Chi Wai Lau, Baohua Liu, Ajay Chawla, Ronald Ching Wan Ma, Yu Huang, Kathy O Lui, Xiao Yu Tian

**Affiliations:** 1 School of Biomedical Sciences, Faculty of Medicine; Chinese University of Hong Kong, Hong Kong SAR, China.; 2 CUHK Shenzhen Research Institute, Shenzhen, China.; 3 Shenzhen Key Laboratory for Systemic Aging and Intervention (SAI), National Engineering Research Center for Biotechnology (Shenzhen), International Cancer Center, Shenzhen University, Shenzhen, China.; 4 Guangdong Key Laboratory of Genome Stability and Human Disease Prevention, Department of Biochemistry & Molecular Biology, School of Basic Medical Sciences, Shenzhen University, Shenzhen, China.; 5 Department of Physiology, Department of Medicine, University of California San Francisco, CA 94143, United States.; 6 Department of Medicine and Therapeutics, Li Ka Shing Institute of Health Sciences, Hong Kong Institute of Diabetes and Obesity, The Chinese University of Hong Kong, Prince of Wales Hospital, Hong Kong, SAR, China.; 7 Department of Chemical Pathology, Li Ka Shing Institute of Health Sciences, Chinese University of Hong Kong, Hong Kong SAR, China.

**Keywords:** Endothelial cell, PPARδ, HIF1α, hindlimb ischemia, vascular homeostasis

## Abstract

**Rationale:** Restoration of vascular perfusion in peripheral arterial disease involves a combination of neovessel formation and the functional restoration of vascular endothelium. Previous studies indicated that ligand-dependent PPARδ activation enhances angiogenesis. However, how PPARδ is triggered by hypoxia and its downstream effects during post-ischemic vascular repair was not well understood.

**Methods:** We induced experimental hindlimb ischemia in endothelial cell selective *Ppard* knockout induced by Cdh5-Cre mediated deletion of floxed Ppard allele in mice and their wild type control and observed blood perfusion, capillary density, vascular relaxation, and vascular leakage.

**Results:** Deletion of endothelial *Ppard* delayed perfusion recovery and tissue repair, accompanied by delayed post-ischemic angiogenesis, impaired restoration of vascular integrity, more vascular leakage and enhanced inflammatory responses. At the molecular level, hypoxia upregulated and activated PPARδ in endothelial cells, whereas PPARδ reciprocally stabilized HIF1α protein to prevent its ubiquitin-mediated degradation. PPARδ directly bound to the oxygen-dependent degradation domain of HIF1α at the ligand-dependent domain of PPARδ. Importantly, this HIF1α-PPARδ interaction was independent of PPARδ ligand. Adeno-associated virus mediated endothelium-targeted overexpression of stable HIF1α *in vivo* improved perfusion recovery, suppressed vascular inflammation, and enhanced vascular repair, to counteract with the effect of *Ppard* knockout after hindlimb ischemia in mice.

**Conclusions:** In summary, hypoxia-induced, ligand-independent activation of PPARδ in ECs stabilizes HIF1α and serves as a critical regulator for HIF1α activation to facilitate the post-ischemic restoration of vascular homeostasis.

## Introduction

Peripheral artery disease (PAD) of the lower limbs is the third leading cause of atherosclerotic cardiovascular disease after coronary artery disease and stroke 1. Critical limb ischemia is the most severe form of PAD which could lead to ulcer, gangrene, and amputation. Although there are effective therapies to lower the cardiovascular risk and to prevent the progression to critical limb ischemia, patients with PAD continue to be under-recognized and undertreated. Many efforts have been made to enhance lower-extremity blood flow via therapeutic angiogenesis for patients with PAD 2. In addition, there has been much interest in the use of stem cell-derived endothelial cells or modification of resident stem cells 3. However, interventions for severely ischemic PAD patients are still very limited besides endovascular procedures and surgeries to rebuild blood flow. It is important to identify critical endogenous regulators and explore approaches to enhance their function in vivo in order to enhance post-ischemic vascular recovery.

To date, several important transcription factors have been identified for post-ischemic vascular recovery, including KLF5, ETS1, COUP-TFII, etc. 4. Many of these transcription factors regulate post-ischemic angiogenesis through expression of growth factors VEGFs, PDGFs, and their receptors. For example, ETV2 mediates VEGFR2 expression and contributes to neovascularization after hindlimb ischemia injury 5. TFEB also facilitates angiogenesis through activation of AMPK and autophagy 6. Importantly, expression of HIF1α after ischemia is a critical event for the induction of angiogenic factors, as well as mobilization of angiogenic cells 7. Reducing HIF1α inactivation could improve angiogenesis in ischemic muscle 8. In addition, the stability of HIF1α is also modulated by many factors, including enzymes such as heme oxygenase-1 and glutaredoxin-1 during post-ischemic angiogenesis 9, 10. Modulating HIF1α activity and identifying its interacting factors could provide some hint for developing therapies to improve perfusion.

The peroxisome proliferator-activated receptors (PPARs) are ligand-activated transcription factors in which three distinct isoforms (PPAR-α, -γ, and -δ) have been identified in tissues. Previous studies demonstrated the protective role of PPARδ agonists in the cardiovascular system against atherosclerosis, stroke, aortic aneurysm, diabetic vasculopathy, etc. 11-13. PPARδ agonist also inhibits vascular inflammation and reduces atherosclerotic lesions in mouse models 11, 14-16. Early studies suggested that PPARδ agonists, such as GW501516 enhance angiogenesis of human endothelial cells in vitro 17. Prostacyclin also promotes the pro-angiogenic function of human endothelial progenitor cells in a PPARδ-dependent manner 18. Likewise, PPARδ agonists enhance the regenerative capacity of human endothelial progenitor cells 19, 20, and also protect endothelial cells from apoptosis 21. These observations suggest that ligand-induced PPARδ activation may play a positive role in vascular homeostasis while the detailed mechanism and regulation is yet to be better understood.

In this study, we have showed that endothelial expression of PPARδ regulates several aspects of vascular homeostasis by enhancing post-ischemic angiogenesis, and endothelial barrier function, while inhibiting endothelial activation and inflammatory responses. We also showed an important role of hypoxia-induced PPARδ, which reciprocally enhances HIF1α stability and its downstream target genes participating in the vascular repair and restoration of vascular integrity. The interaction and regulation of PPARδ-HIF1α is critical for perfusion recovery in hindlimb ischemia.

## Methods

### Animals

All the mice were housed at 22 °C in a barrier facility and kept on a 12-hour light, 12-hour dark cycle with free access to food and water. The *Ppard* floxed mutant mice (B6.129S4-*Ppard^tm1Rev^*/J) and the VEC-cre transgenic mice (B6;129-Tg*(Cdh5-cre)^1Spe^*/J) were originally from Jackson laboratory. Both strains were backcrossed with C57BL/6 mice before they were crossed to generate endothelial cells specific deletion of *Ppard* as *Ppard*^f/f^;*Cdh5*^Cre/+^ (*Ppard*^EC-KO^) mice. Their wild type controls were *Ppard*^f/f^ (*Ppard*^EC-WT^) mice. Mouse genotype were validated by DNA genotyping using Jax protocols, mRNA and protein expression. All the experiments were performed using littermates, which were randomized to experimental groups. The observers of mouse experiments and analysis were blinded with genotype information, which was matched afterwards.

### Hindlimb ischemia model and assessments

Hindlimb ischemia (HLI) was induced by ligation of femoral artery in male mice at 10-12 weeks of age. Mice were anesthetized via intraperitoneal injection of a combination of 75 mg/kg ketamine and 10 mg/kg xylazine (Alfasan Co, Netherlands) before the unilateral ligation was performed. In this unilateral ischemia model, the contralateral limb was considered as a control. Mice were kept warm on a heatpad at 36 ± 1.0 °C during the procedure. Blood perfusion was measured by imaging of plantar regions of interests with Laser Doppler Imager (Moor Instruments) and the average lower leg blood flow was presented as the ratio of ischemic to non-ischemic side at days 0, 3, 7, 14, 21 and 28 following HLI. Vasculature imaging of the thigh was performed with the Laser Speckle Contrast Imaging System RFLSI III (RWD Life Science Co.).

### Histological analysis

GA muscle was embedded in OCT and frozen in cooled 2-methylbutane. Frozen section was cut at 10 µm. Sections were fixed with 4% paraformaldehyde, washed in PBS, and stained with hematoxylin and eosin. Some sections were fixed in Mordant in Bouin's solution for 30 min, stained sequentially with Weigert's iron hematoxylin, Biebrich scarlet-acid fuchsin, phosphotungstic/phosphomolybdic acid, and aniline blue. Sections were washed, dehydrated, and mounted with a xylene-based mounting medium.

### Functional assay by wire myograph

After mice were euthanized by CO_2_ inhalation, femoral arteries were removed and placed in oxygenated ice-cold Krebs solution that contained (mmol/L) 119 NaCl, 4.7 KCl, 2.5 CaCl_2_, 1 MgCl_2_, 25 NaHCO_3_, 1.2 KH_2_PO_4_, and 11 D-glucose. Changes in isometric tone of the femoral arteries were recorded in wire myograph (Danish Myo Technology, Aarhus, Denmark). The vascular segments were stretched to an optimal baseline tension of 0.8-1 mN and then allowed to equilibrate for 1 h before the experiment commenced. Segments were first contracted with 60 mmol/L KCl and rinsed in Krebs solution. After several washouts, phenylephrine (10 μmol/L) was used to produce a steady contraction, acetylcholine (10 nmol/L to 30 μmol/L) was added cumulatively to induce endothelium-dependent vasodilatation on different segments. Endothelium-independent vasodilatation to SNP was performed in the presence of nitric oxide synthase inhibitor L-NAME (0.1 mmol/L), indomethacin (1 μmol/L), and 20 mmol/L KCl. Statistical significance was calculated either using the area under curve for each segment or indicated on the individual data points.

### RNA isolation and quantitative PCR analysis

Total RNA was extracted from cells or mouse tissues using TRIzol reagent RNAiso Plus (Takara, cat# 9109) and 1 μg of total RNA was reverse transcribed into complementary DNA (cDNA) using 5× PrimeScript RT Master Mix (Takara, cat# RR036A), following the manufacturer's instructions. The mRNA levels were determined by quantitative PCR with TB Green® Premix Ex Taq™ (Tli RNase H Plus (Takara, cat# RR420A) detected on an Applied Biosystems ViiA7. All primer sequences are listed in **Table S1**.

### Flow cytometric analysis

At 7 days after HLI surgery, GA muscle from the injured leg was digested with 800 U/ml Collagenase IV + 1 U/ml Neutral Protease (both from Worthington Biochemical) for 60 min. The cells were then suspended in FACS buffer (2% FBS with 2 mmol/L EDTA in PBS), and filtered through 40-μm strainer (BD Biosciences) to generate single-cell suspensions. Cells were firstly incubated with LIVE/DEAD Aqua (Thermo) for viability following manufacturer's protocol together with anti-CD16/CD32 (10 µg/mL, Biolegend) for 30 min. For flow cytometric analysis, cells were then incubated with fluorescent-conjugated anti-mouse antibodies listed in the **Table S2**. Endothelial cells are defined as CD45^-^CD31^+^CD144^+^. Macrophages are first gated on CD45^+^Ly6G^-^CD11b^+^, and further separated as tissue macrophages (F4/80^+^Ly6C^lo^), and monocyte-derived macrophages (F4/80^mid^Ly6C^hi^). Cells were fixed with 1.6% paraformaldehyde for 30 min at 4 °C until further analysis using FACSAria Fusion (BD). Data were analyzed using FlowJo.

### Immunofluorescence staining

Frozen sections of GA muscle were then fixed in acetone, blocked with normal goat or donkey serum (Abcam), and incubated with primary antibodies and appropriate fluorescence-conjugated secondary antibodies, followed by Hoechst 33342 (Thermo) for nucleus, and mounted in fluorescence mounting medium (Electron Microscopy, Cat#17985-10). Detailed information of all the antibodies used can be found in **Table S2**.

### Lectin injection for vascular structure

After HLI 14 days, functional vessels were stained with fluorescein isothiocyanate (FITC) - Griffonia simplicifolia lectin I (Vector Laboratories, cat#FL-1101-5) (100 μg/mL in PBS) via tail vein injection. Mice were euthanized 5 min after injection and perfused through the heart with PBS followed by 4% paraformaldehyde in PBS. The gastrocnemius muscle was processed for immunofluorescence staining.

### Aortic ring assay

Mouse aortic ring assay was performed as previously described 22. Briefly, the thoracic aortic rings were isolated and 1-mm long aortic rings were embedded in growth factor-reduced Matrigel supplemented with 20 U/mL heparin. The aortic rings were then cultured in Opti-MEM supplemented with 2.5% FBS and 30 ng/mL hVEGF (Peprotech).

### Cell culture and cell transfection

Mouse brain microvascular endothelial cells (BMECs, from Angio-Proteomie) were transfected with mouse *Ppard* siRNA (ThermoFisher siRNA ID#151214, #151213), mouse *Hif1a* siRNA (ThermoFisher siRNA ID#158953, #158954) or universal scrambled negative control siRNA using Lipofectamine RNAiMAX Transfection Reagent (Invitrogen), according to the manufacturer's instructions.

### Tube formation assays

To examine the effect of *Ppard* knockout on the in vitro angiogenesis of bone marrow derived endothelial cells (BM-ECs). Briefly, a 96-well plate was coated with growth factor-reduced Matrigel (Corning, cat# 354230), which was allowed to solidify at 37 °C for 30 min. BM-ECs (1.8×10^4^ per well) were seeded and cultured in EGM-2 medium (Lonza, cat# CC-3202) with 10% FBS under normoxia or hypoxia. The tube-like networks were photographed under a microscope (IX83, Olympus). The perimeters of all the tubes were measured for semi-quantitative analyses using ImageJ Angiogenesis analyzer plug-in as previous described 23.

### Plasmids/Transfection

Plasmids transfection was performed by Lipofectamine™ 3000 Transfection Reagent (Thermo Fisher) following the manufacturer's instructions in HEK293T cells and Hela cells. HA-HIF1alpha-pcDNA3 (HA-HIF1α in short, Addgene plasmid #18949; http://n2t.net/addgene:18949; RRID: Addgene_18949), and HA-HIF1alpha P402A/P564A-pcDNA3 (Addgene plasmid # 18955; http://n2t.net/addgene:18955; RRID: Addgene_18955) were gifts from William Kaelin 24. HIF1alpha(401delta603)_R27G (HA-ΔODD-HIF1α in short) was a gift from Eric Huang (Addgene plasmid #52215; http://n2t.net/addgene:52215; RRID: Addgene_52215) 25. HRE-luciferase (HRE-Luc in short) was a gift from Navdeep Chandel (Addgene plasmid #26731; http://n2t.net/addgene:26731; RRID: Addgene_26731) 26. Plasmid pcDNA3.1 Flag-HIF1B (#930) was a gift from James Brugarolas (Addgene plasmid #99916; http://n2t.net/addgene:99916; RRID: Addgene_99916) 27. Human *PPARD* was initially amplified from cDNA template and then cloned into p3XFLAG-CMV™-10 (Sigma, E7658) fused with Flag or HA tag. To generate the truncated domains of *PPARD* (Flag-DBD and Flag-LBD) as previously described 28, relevant fragments were amplified by indicated primers listed in **Table S1** and then all were cloned into p3XFLAG-CMV™-10.

### Protein extraction and Western blotting

Cells were lysed in 1X SDS lysis buffer [50 mmol/L Tris-HCl (pH 6.8), 100 mmol/L DTT, 2% SDS, 0.1% bromophenol blue, 10% glycerol] and then boiled for 10 min. Standard Western blotting analyses were performed. Lysates in DTT-containing SDS sample buffer were separated in 8% or 12% SDS-polyacrylamide gels and transferred to PVDF transfer membranes (Thermo) and incubated with primary antibodies including anti-HIF1α, anti-PPARδ, and anti-GAPDH (antibodies diluted concentration following the manufacturer's instructions). Expression was then detected with BioRad ChemDoc MP Imaging System using blotting-grade HRP conjugate (Bio-rad) and Immobilon Western Chemiluminescent HRP Substrate (Millipore) for chemiluminescent detection. All antibodies are listed in **Table S2**.

### Luciferase reporter assay

5 × 10^4^ per well of HEK293T cells were seeded in 24 well plates and transfected with plasmids of 250 ng HRE-Luc, 25 ng pRL-CMV Renilla (Rluc) and with or without 250 ng Flag-PPARδ. Luciferase activity was measured after 24 h by dual luciferase assay following the manufacturer's instructions (Promega, USA). The relative luciferase activity was determined by firefly luciferase value versus renila luciferase value. The presented data showed the fold change normalized to control group.

### HIF1α half-life assays

The BMECs were exposed to hypoxia (1% O_2_) for 4 h after transfected with *Ppard* siRNA for 48 h. Cells were exposed to 50 μg/mL cycloheximide (Sigma, cat#01810) for the indicated time to block protein synthesis. The cells were collected for Western blotting.

### Chromatin Immunoprecipitation analysis (ChIP)

ChIP assay was carried out according to the previously published method 29. Briefly, Hela cells were lyzed in ChIP-IP buffer (150 mmol/L NaCl, 50 mmol/L Tris-HCl (pH 7.5), 5 mmol/L EDTA, NP-40 (0.5% vol/vol), Triton X-100 (1.0% vol/vol)) with addition of protein inhibitors (Sigma, cOmplete™ Protease Inhibitor Cocktail). After sonication, centrifugation and protein A/G agarose beads pretreatment (Pierce™ Protein A/G Agarose, #20421), clear supernatant was incubated with anti-HIF1α primary antibody for 6 h and then immunoprecipitates were captured by protein A/G agarose beads for another 4 h incubation. Finally, the chromatin DNA was eluted in 10% (wt/vol) Chelex 100 slurry ((Bio-Rad, #142-1253) by boiling for 10 min.

### Immunoprecipitation

Cell lysates were prepared in IP lysis buffer (20 mmol/L Tris-HCl (pH 7.9), 200 mmol/L NaCl, 5 mmol/L MgCl_2_, 10% glycerol, 0.2 mmol/L EDTA, and 0.1% NP-40) supplemented with protease and phosphatase inhibitors (Sigma, complete™ Protease Inhibitor Cocktail and PhosSTOP™). Clear cell supernatant was incubated with the respective anti-Flag or HA agarose beads for 4 h at 4 °C. After the beads were washed with IP lysis buffer three times, the immunoprecipitates were eluted in Laemmli sample buffer and subjected to western blotting analysis.

### Evans blue staining

After HLI at day 10, mice were injected with 300 µL of 1% Evans Blue (in PBS) via the tail vein. The dye was allowed to circulate in vivo for 30 min, followed by cardiac perfusion with PBS (10 mL per mouse). Quadriceps were isolated and weighed, and the dye was extracted overnight with deionized formamide (1 mL per muscle) and measured at an optical density of 610 nm.

### FITC dextran extravasation

After HLI at day 7, mice were injected with 50 μL 25 mg/mL 70 kDa FITC dextran (Sigma, cat# FD70S) via the tail vein 30 min before sacrifice. Microscopic visualization of FITC dextran extravasation was performed on OCT-embedded tissue sections and co-stained with anti-CD144 antibody (Invitrogen).

### Adeno-associated viruses (AAV) administration

For AAV-mediated *Icam2*-driving *HIF1A*-P402A/P564A (M1 in short) or *HIF1A*-(ΔODD)/R27G (ODD domain deleted, M2 in short) overexpression, the M1 and M2 viruses (total 10^11^ vg in 30 μL was injected into the hindlimb muscles of both sides 1 week before HLI.

### Statistical analysis

All data were presented as means ± SEM and the numbers of independent experiments are indicated. Flow cytometry data were analyzed by FlowJo. Western blot images were analyzed by ImageJ. Student's t test was used for comparison between two samples, and one-way ANOVA and multiple comparison test was used for more than two samples in GraphPad Prism. * p < 0.05, ** p < 0.01, and *** p < 0.001 was indicated as statistically significant.

## Results

### Selective deletion of endothelial *Ppard* impairs vascular perfusion in mice after hindlimb ischemia

**Figure 1:**
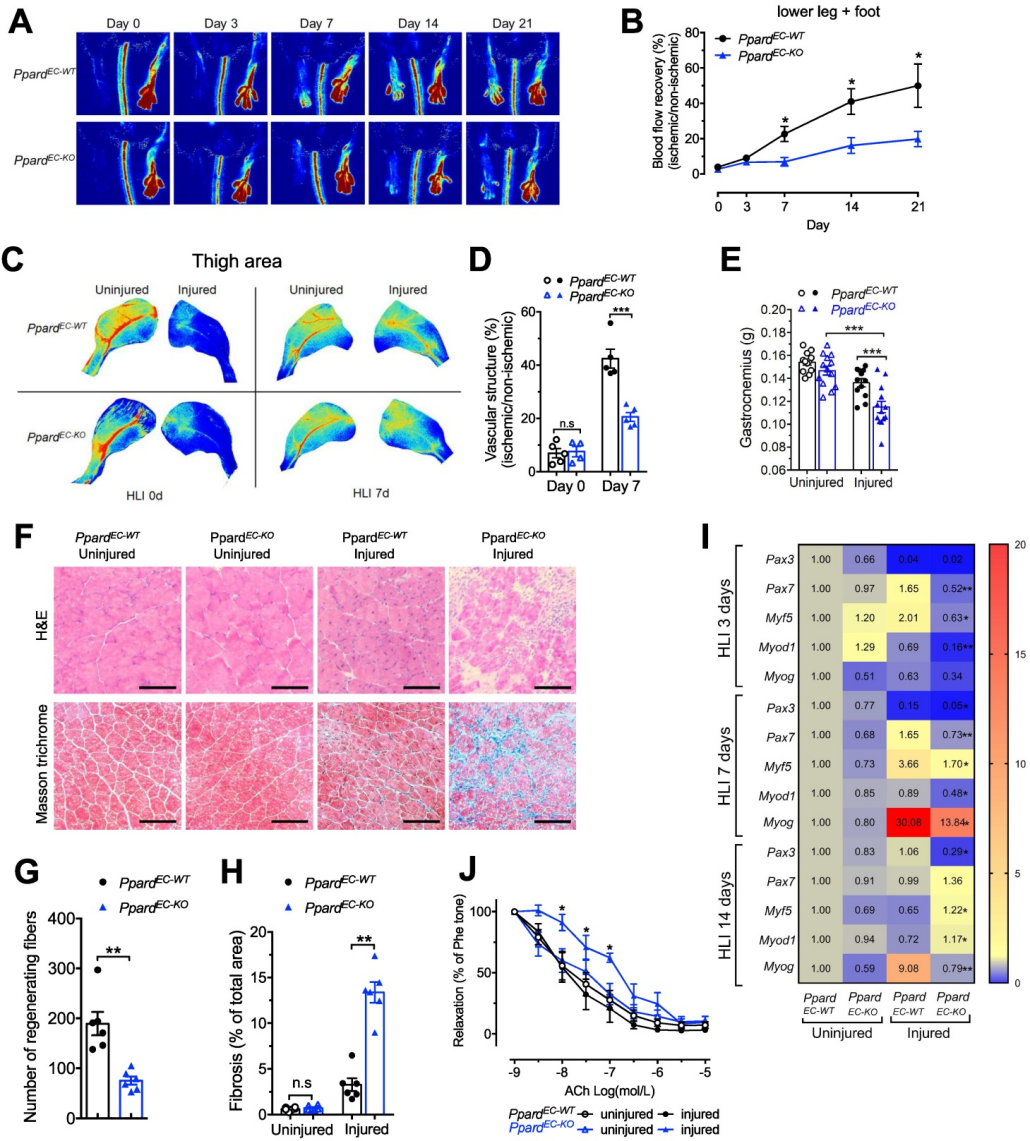
** Endothelial deletion of *Ppard* impairs vascular remodelling and damages skeletal muscle regeneration.** Perfusion imaging following HLI, showed in representative images (**A**) and analysis in (**B**) (n = 6 mice for each group). **C-D**, Representative images of Vasculature imaging in leg area were recorded at days 0 and 7 in representative images (**C**) and summarized analysis in (**D**) (n = 5, each group).** E**, Muscle weights of gastrocnemius (GA) were measured at days 14 after HLI. **F**, Haematoxylin/eosin staining (up) and Masson trichrome staining (bottom) at days 14 after HLI (n = 6, each group). Scale bar, 100 μm. **G**, Quantification of regenerating muscle fibers based on H&E staining. **H,** Quantification of fibrosis (blue colour) based on trichrome staining. **I**, Heatmap of qPCR data at days 3, 7, 14 after HLI. The number in each cell represents the fold change compared with *Ppard*^EC-WT^ uninjured GA (n = 5-6, each group). Results are means ± SEM. **J**, Concentration-response curves to acetylcholine (ACh) in femoral arteries at days 28 after HLI. Results are means ± SEM. * p < 0.05, ** p < 0.01, *** p < 0.001 between groups or vs injured *Ppard*^EC-WT^. Student's t test was used for comparison between two samples, and one-way ANOVA and multiple comparison test was used for more than two samples.

After generating the endothelial selective *Ppard* knockout *Ppard*^EC-KO^ and wild type *Ppard*^EC-WT^ mice, the knockout efficiency was tested in endothelial cells (ECs) from several organs showed diminished *Ppard* mRNA expression and protein expression (Figure S1A, B). Vascular perfusion measurement after hindlimb ischemia (HLI) showed that recovery of perfusion was reduced in the *Ppard*^EC-KO^ mice over a period of 3 weeks (Figure 1A-B). Similar results were also obtained by imaging of vascular structure at 7 days after HLI (Figure 1C-D). At the end of 3 weeks, toenail necrosis as one of the parameters of ischemic score 30, could still be observed in the *Ppard*^EC-KO^ mice, reflecting an impaired recovery (Figure S1C-D). The gastrocnemius (GA) muscle became smaller after HLI which was further reduced in the *Ppard*^EC-KO^ mice (Figure 1E), with less regenerating muscle fiber with centralized nuclei, and more scar formation replacing muscle fiber in the GA of *Ppard*^EC-KO^ mice (Figure 1F-H). Delayed muscle repair in the *Ppard*^EC-KO^ mice was also observed from the gene profiling which showed the delayed upregulation of muscle progenitor markers *Pax3* and *Pax7*; early myogenic markers *Myf5*, *Myod1*, and *Myog*, shifting from normally 3 and 7 days to a later time point at 14 days (Figure 1I). In addition, the endothelium-dependent vasodilatation induced by acetylcholine (Figure 1J) was impaired after HLI in the femoral arteries from *Ppard*^EC-KO^ mice, indicating sustained impairment of endothelial function; whereas nitric oxide (NO) donor sodium nitroprusside (SNP) induced vasodilatation was similar, indicating the smooth muscle function was less affected (Figure S1E). These results suggested a delayed structural and functional recovery after HLI due to loss of endothelial PPARδ.

### Deletion of endothelial *Ppard* impairs post-ischemic angiogenesis

**Figure 2:**
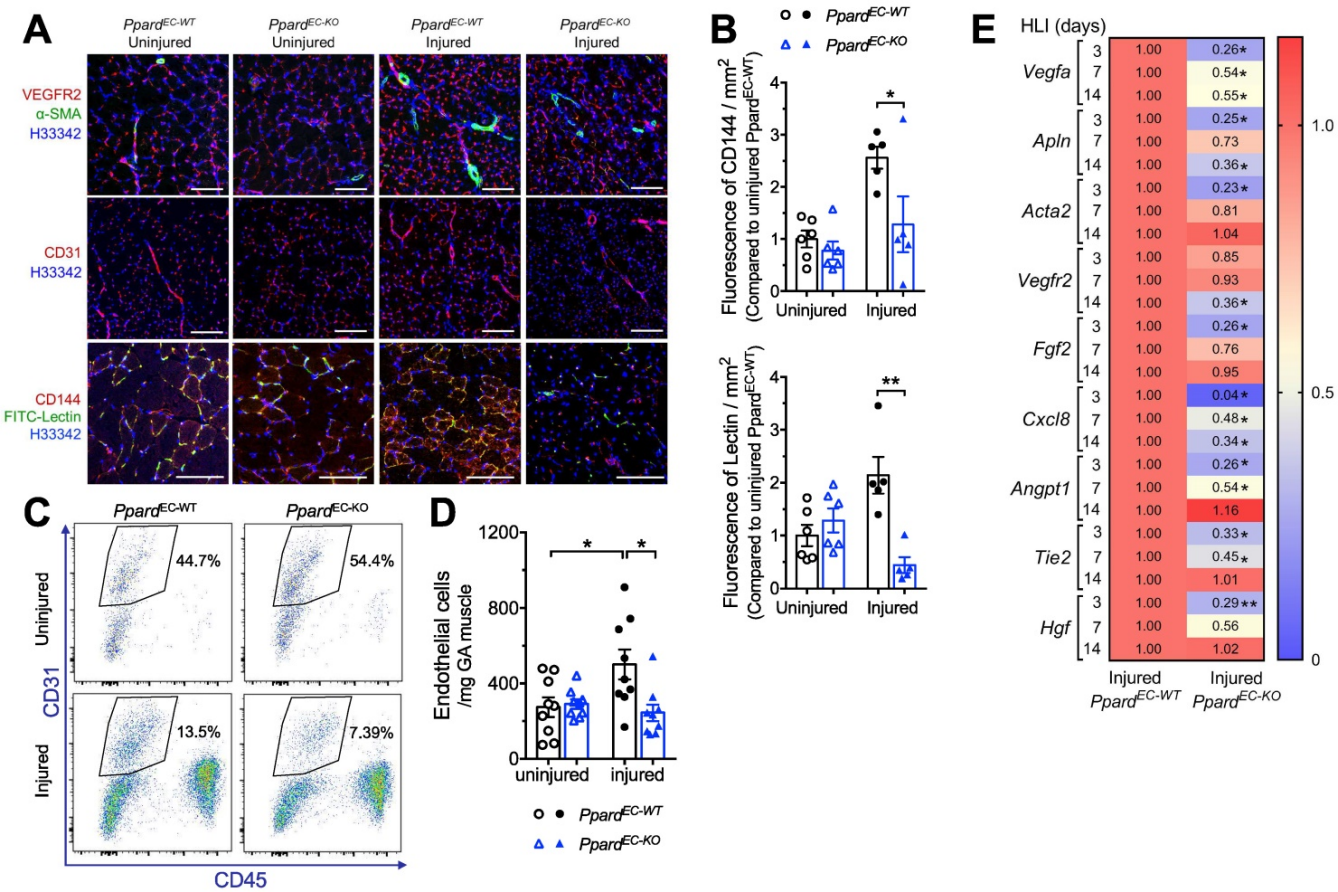
** Deletion of endothelial *Ppard* impairs post-ischemic angiogenesis. A**, Muscle frozen sections stained with VEGFR2, CD31 and CD144 for ECs, a-SMA for arteriole and FITC-lectin for functional vessel (n = 6, each group). Scale bar: 200 μm. **B**, Analysis of FITC-lectin to identify functional microvessels and CD144 to identify ECs at days 14 after HLI (n = 6, each group) for **Figure 3A**. **C** (representative flow plots) and **D** (summarized analysis) of CD45^-^CD31^+^ ECs at day 3 after HLI. **E**, Heatmap of the qPCR data for angiogenesis related genes at indicated time after HLI. The number in each cell represents the fold change compared with *Ppard*^EC-WT^ injured GA (n = 5-6, each group). Results are means ± SEM. * p < 0.05, ** p < 0.01 between groups. Student's t test was used for two samples, and one-way ANOVA and multiple comparison test was used for more than two samples.

Because PPARδ ligands stimulate angiogenesis in endothelial cells 17-20, although the endothelial deletion of Ppard was not fully characterized, we first examined whether post-ischemic angiogenesis was affected by EC selective deletion of PPARδ. Immunofluorescence showed that the post-ischemic increase of VEGFR2, as well as CD31, was less in the *Ppard*^EC-KO^ mice (Figure 2A). Expression of α-SMA showing the arterioles also increased less in the *Ppard*^EC-KO^ mice (Figure 2A). Notably, FITC-lectin which labels the functional endothelium showed diminished signals in the *Ppard*^EC-KO^ mice (Figure 2A-B). Flow cytometric analysis demonstrated less increase of EC labeled as CD45^-^CD31^+^ cells in *Ppard*^EC-KO^ mice (Figure 2C-D). Similarly, qPCR analysis of mRNA expression suggested several angiogenic factors such as *Vegfa*, *Fgf2*, did not increase similar as wild type after ischemia, while the induction of some angiogenic factors such as Angpt1 and Tie2 were delayed (Figure 2E). In vitro, the aortic segments from the *Ppard*^EC-KO^ mice also showed less sprouting (Figure S2A-B). These results suggested impaired post-ischemic hypoxia-induced angiogenesis due to the loss of PPARδ in ECs.

### Deletion of endothelial *Ppard* exacerbates vascular hyperpermeability

**Figure 3:**
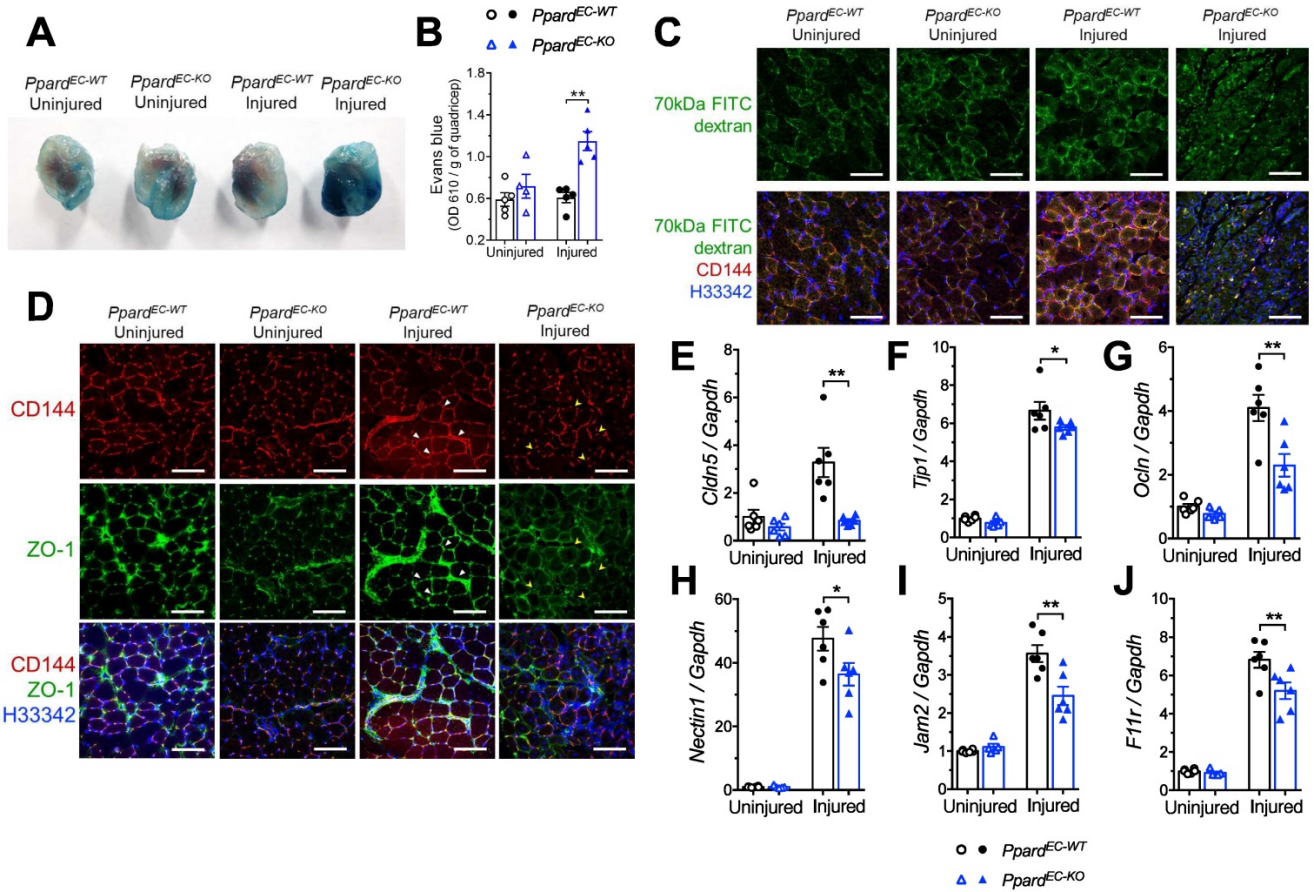
** Deletion of endothelial *Ppard* increases vascular permeability. A** (Representative images) and **B** (quantification) of Evans Blue in quadriceps 14 days after HLI. **C**, Representative images of FITC-labeled 70 kDa dextran co-stained with CD144 in GA at day 7 after HLI (n = 5, each group). Scale bar: 200 μm. **D**, Representative images of ZO-1 to co-localize with CD144 in GA at day 10 after HLI (n = 6, each group). Scale bar: 200 μm. Triangle indicates continuous endothelium. Arrowhead indicates discontinuous endothelium. **E-J**, qPCR analysis of muscles 3 days after HLI (n = 6, each group). Results are means ± SEM. * p < 0.05, ** p < 0.01 between groups by one-way ANOVA and multiple comparison test.

Endothelial barrier function is an important aspect indicating the functional restoration of injured vasculature 31. Whether endothelial barrier function is regulated by PPARδ is unknown. To examine vascular barrier integrity, we first showed that a lot more albumin leakage surrounding the capillaries after HLI was found in the *Ppard*^EC-KO^ mice, which is a parameter to assess vascular hyperpermeability (Figure S3). This change was also quantified by Evans blue, a dye binding to serum albumin which also showed more leakage into the injured muscles from *Ppard*^EC-KO^ mice (Figure 3A-B). We then used FITC labelled 70-kDa dextran administration to examine the EC junctional alterations, in which the aggregated FITC at capillaries indicates increased extravasation in the injured muscles from *Ppard*^EC-KO^ mice which was persistent after HLI (Figure 3C). Two junctional proteins CD144 and tight junction ZO-1, which are functionally important in ECs, also became more discontinuous in the newly emerged capillaries of *Ppard*^EC-KO^ mice (Figure 3D, indicated by yellow arrowheads), which was more continuous in the capillaries from *Ppard*^EC-WT^ mice (Figure 3D, indicated by white triangles). In addition, the upregulation of several genes including *Cldn5*, *Tjp1* (ZO-1), *Ocln* (occludin), which are involved in tight junction (Figure 3E-G); as well as *Nectin1*, *F11r* (Jam-A), and *Jam2* (Jam-b) which are involved in adherens junction and endothelial leukocyte adhesion (Figure 3H-J), were attenuated in the injured muscle from *Ppard*^EC-KO^ mice. These results indicated impaired restoration of endothelial barrier function due to the loss of PPARδ after HLI.

### Deletion of endothelial *Ppard* promotes endothelial activation and inflammatory responses

**Figure 4:**
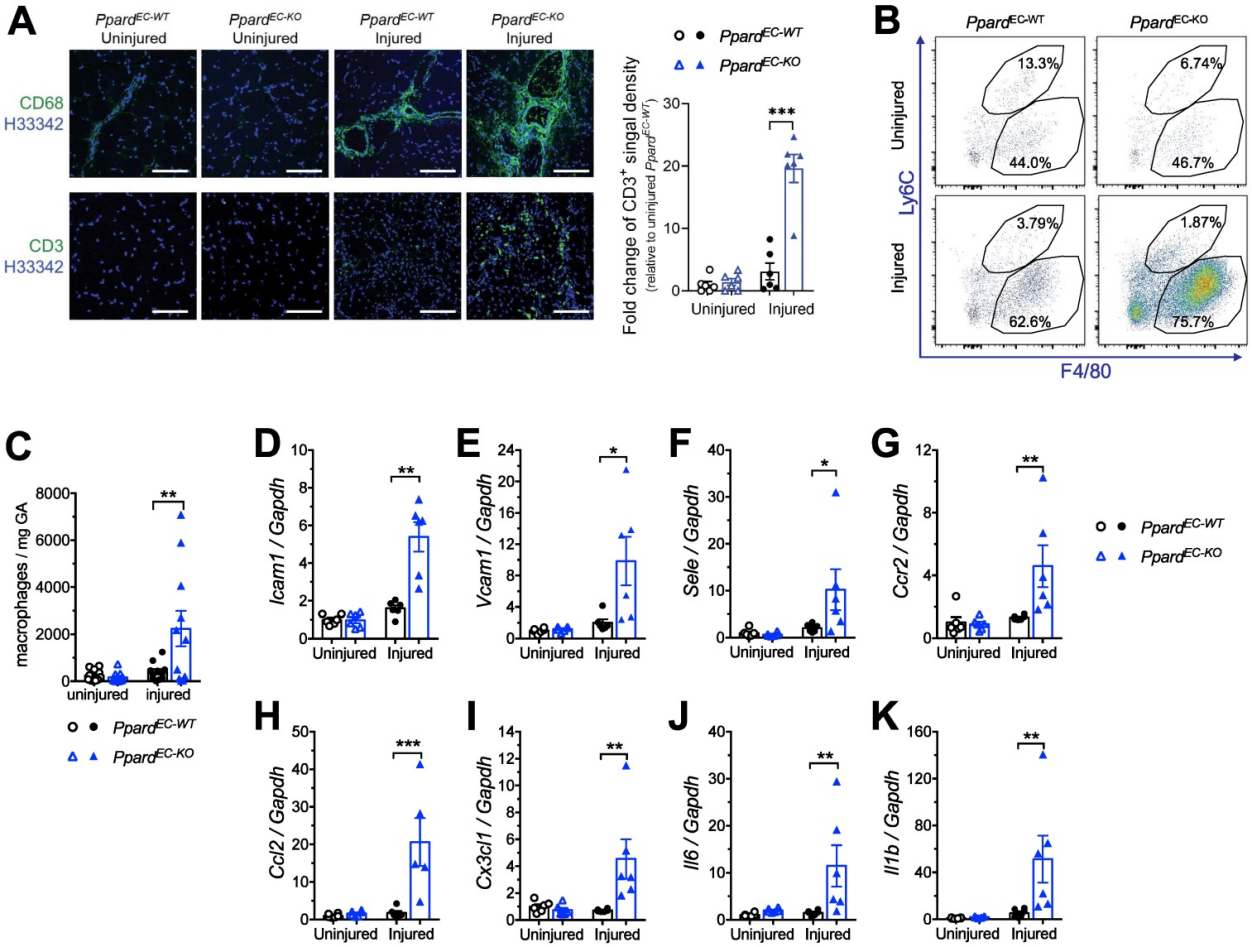
** Deletion of endothelial *Ppard* enhances endothelial activation and inflammatory responses. A**, Representative images of immunofluorescence showing CD68 staining (upper) and CD3 (lower) 14 days after HLI (n = 5, each group). Scale bar: 200 μm. **B** (Representative flow plots) and **C** (summarized analysis) of F4/80^+^Ly6C^hi^ monocyte/macrophages and F4/80^+^Ly6C^low^ macrophages at day 10 after HLI. **D-K**, qPCR analysis for vascular inflammatory markers Vcam1, Icam1 and E-selectin in GA 14 days after HLI (n = 6, each group). Results are means ± SEM. * p < 0.05, ** p < 0.01 between groups by one-way ANOVA and multiple comparison test.

Because impaired endothelial integrity promotes endothelial activation, we characterized vascular inflammatory responses after HLI. Immunofluorescence showed that macrophage and T lymphocyte infiltrations were increased in the ischemic muscle from *Ppard*^EC-KO^ mice (Figure 4A). More accumulation of tissue macrophages (labeled as F4/80^+^Ly6C^lo^), which was likely due to the infiltration of monocyte-derived macrophages (F4/80^mid^Ly6C^hi^) was also observe by flow cytometric analysis (Figure 4B-C). Consistently, many vascular inflammatory factors including adhesion molecules *Icam1*, *Vcam1* and* Sele (E-selectin)* (Figure 4D-F), chemokine and their receptors *Ccr2*, *Ccl2*, *Cx3Cr1* (Figure 4G-I), and cytokine *Il1b* and* Il-6* (Figure 4J-K), also remained at high level in the ischemic muscle from *Ppard*^EC-KO^ mice. These results indicated that loss of PPARδ in ECs caused a persistent endothelial activation and unresolved chronic inflammation after HLI.

### PPARδ enhances HIF1α activity in endothelial cells in response to hypoxia

**Figure 5:**
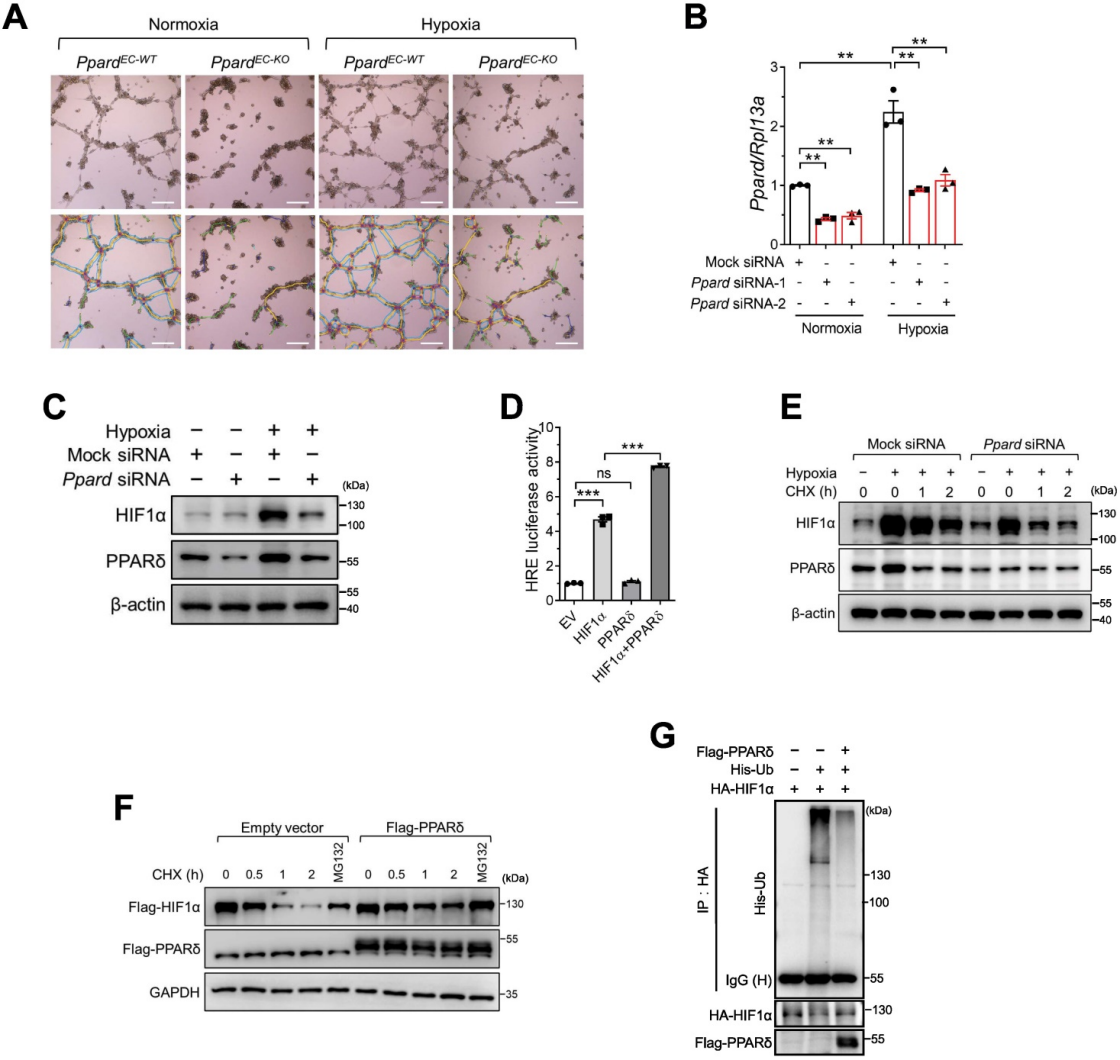
** PPARδ enhances HIF1α activity in endothelial cells in response to hypoxia. A** (representative images) and **B** (summarized analysis) of tube formation of bone marrow-derived endothelial cells on matrigel under normoxia and hypoxia, analyzed using the Angiogenesis Analyzer of Image J (n = 4, each group). Scale bar: 100 μm. **C**, Immunoblots showing HIF1α treated with Ppard siRNA in mBMECs under hypoxia for 12h. **D**, Luciferase reporter assay showing HRE-luc activity in HEK293T cells transfected with indicated plasmids. EV: empty vector. **E**, Immunoblots showing the effect of CHX 50 μg/mL at indicated time after hypoxia for 4 h. **F**, Immunoblots in HEK293T following indicated treatments under normoxia. CHX, 50 µg/mL. MG132, 10 µmol/L. **G**, In vivo ubiquitination assay showing the ubiquitinated HIF1α levels in HEK293T after transfection of indicated plasmids. Representative data have at least three biological replicates. Results are means ± SEM. * p < 0.05, ** p < 0.01 between groups by one-way ANOVA and multiple comparison test.

During ischemic injury, endothelium is exposed to hypoxia. To examine the response of ECs to hypoxia, we first used bone marrow -derived endothelial progenitor cells (EPCs) which are capable of angiogenesis in vitro, rather than using primary ECs from muscle due to the difficulty of maintaining primary EC phenotype in vitro. We observed that tube formation enhanced by hypoxia was impaired in *Ppard*^EC-KO^ EPCs (Figure 5A, analysis in Figure S4A-D). In response to hypoxia, hypoxia-inducible factor (HIF1α) is activated and induces downstream gene expression for vascular regeneration and remodeling in endothelial cells 32. Therefore, we wondered whether PPARδ might modulate HIF1α activity in regulating EC function. Hypoxia upregulated PPARδ mRNA (Figure 5B) and protein (Figure 5C) expression in BMECs. Meanwhile, *Ppard* siRNA treatment attenuated hypoxia-induced upregulation of HIF1α protein in BMECs (Figure 5C). However, HIF1α mRNA expression was not affected by *Ppard* siRNA (Figure S4E). In addition, we observed the hypoxia-induced upregulation of several well-known HIF1α target genes such as *Vegfa*, *Vegfr2*, *Pdk1,* as well as *Angptl4*, the common target gene of both PPARδ and HIF1α, were attenuated by silencing of *Ppard* in BMECs (Figure S4F-I). We then asked whether PPARδ might be directly involved in HIF1α-mediated transactivation. As expected, co-expression of PPARδ with HIF1α enhanced the hypoxia responsive element (HRE) -driven luciferase activity, whereas PPARδ alone had minimal effect (Figure 5D). Furthermore, ChIP assay showed that there was less HIF1α occupancy at the HRE region of *GLUT1*, a well-characterized HIF1α target gene 33, after silencing of *PPARD* in Hela cells (Figure S4J-K). These results suggested that PPARδ underlines HIF1α transactivation.

We thus asked how PPARδ regulated HIF1α transactivation. Because *HIF1A* mRNA was unaffected by Ppard siRNA, we asked whether PPARδ regulates HIF1α protein stability. First, we performed cycloheximide (CHX) chase assay and found a significant decline in HIF1α protein stability in the BMECs after silencing *Ppard* (Figure 5E). Conversely, overexpressing PPARδ stabilized ectopic HIF1α (Figure 5F). Because HIF1α protein degradation is largely dependent on the ubiquitin-proteasome system 34, proteasome inhibitor MG132 was sufficient to restore HIF1α protein in the presence of CHX (Figure 5F)**.** We therefore wondered whether PPARδ modulates HIF1α ubiquitination. As determined by *in vivo* ubiquitination assay, ubiquitinated HIF1α declined notably with PPARδ overexpression (Figure 5G). Together, these results suggested that PPARδ stabilizes HIF1α via inhibiting ubiquitination dependent proteasome-mediated degradation.

### PPARδ interacts with HIF1α in endothelial cells

**Figure 6:**
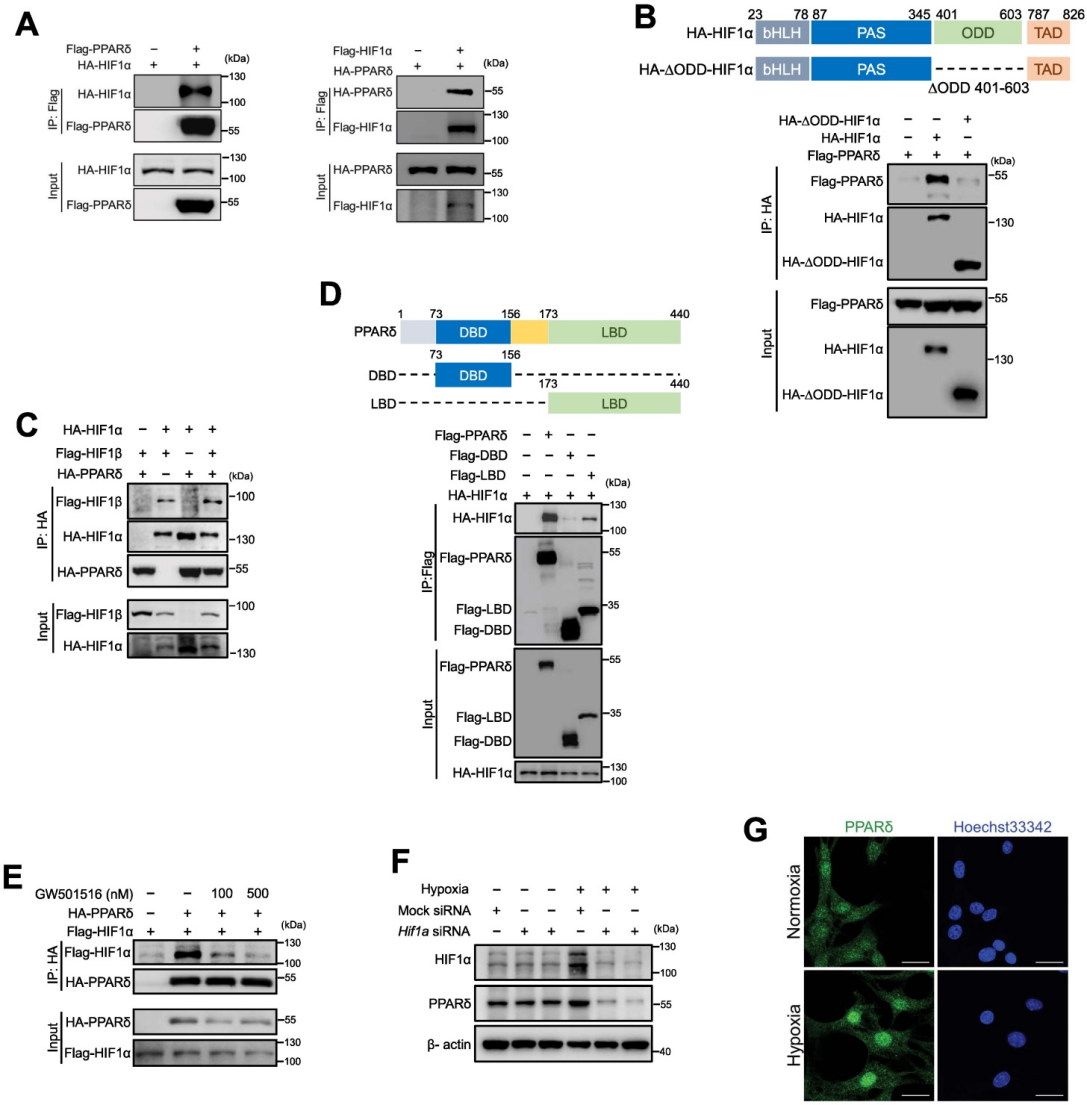
** Hypoxia-induced PPARδ interacts with HIF1α in endothelial cells. A**, Immunoblots showing HIF1α and PPARδ in anti-Flag immunoprecipitates in HEK293T transfected with indicated plasmids. **B**, Schematic diagram showing site of ∆ODD in HIF1α gene and immunoblots showing the Flag-PPARδ in the anti-HA immunoprecipitates from cells co-expressing Flag-PPARδ and full-length HA-HIF1α or Flag-PPARδ and ODD domain deleted HIF1α (HA-∆ODD-HIF1α). **C**, Immunoblots showing the anti-HA immunoprecipitates in HEK293T with transfection of indicated plasmids. **D**, Schematic diagram showing the position of full-length and truncated PPARδ DBD and LBD, and immunoblots showing the anti-Flag immunoprecipitates in HEK293T with indicated plasmids transfected. **E**, Immunoblots showing the anti-HA or anti-Flag immunoprecipitates in HEK293T with indicated plasmids transfected to show the interaction of PPARδ and HIF1α treated with GW501516 (6 h) or solvent control. **F**, Immunoblots of protein expression after transfection with *Hif1a* siRNA in mBMECs after hypoxia for 12 h. **G**, Representative immunofluorescence of PPARδ localization in the nuclei of mBMECs after hypoxia for 12 h (n = 4 biological replicates of each group). Scale bar, 20 μm. All the siRNA transfections were performed with lipofectamine RNA iMax for 48 h, and all the plasmids were transfected with lipofectamine for 36 h, before other treatments. Representative data have at least three biological replicates (**A-F**).

We reasoned that PPARδ was less likely to act on HIF1α by directly writing or erasing any post-translational modifications of HIF1α protein. Because previous study showed that HIF1α could be stabilized by forming complex with co-factors, such as c-Jun, to mask the oxygen dependent degradation (ODD) domain, preventing HIF1α from ubiquitination-dependent degradation 35, we wondered whether PPARδ acts through a similar mechanism. Firstly, co-immunoprecipitation showed that HA-HIF1α or HA-PPARδ existed in the immuneprecipitate of anti-Flag-PPARδ or anti-Flag-HIF1α (Figure 6A), suggesting the interaction between PPARδ and HIF1α. Next, PPARδ failed to bind with ODD-deleted HIF1α, indicating that PPARδ directly occupies the ODD domain of HIF1α to prevent its degradation (Figure 6B). In addition, PPARδ enhanced the formation of HIF1α/β heterodimer (Figure 6C), which is crucial to for HIF1α transactivation. However, the direct interaction of HIF1β with PPARδ was nearly undetectable (Figure S5A). Collectively, our results suggested that PPARδ acts as a co-factor stabilizing HIF1α transcriptional complexes.

### Hypoxia induces ligand-independent activation of PPARδ

PPARδ has an N-terminal DNA-binding domain (DBD) and a C-terminal ligand-binding domain (LBD) for ligand-induced transactivation 28. We further investigated which domain was required for HIF1α binding. As shown by co-IP, HIF1α had a strong affinity to both full-length, and LBD, but not DBD, suggesting LBD underlies the recruitment of HIF1α (Figure 6D). We thus wonder whether the interaction of HIF1α and PPARδ relies on its ligand. Interestingly, PPARδ agonist GW501516 does-dependently counteracted the binding of HIF1α to PPARδ (Figure 6E), indicating that the interaction between HIF1α and PPARδ under hypoxia was most likely independent of PPARδ ligand. GW501516 did not increase HIF1α protein (Figure S5B) or its target genes such as Vegfa under hypoxia in BMECs (Figure S5C-D), whereas PPARδ target gene Pdk4 was induced by GW501516, and also enhanced by hypoxia (Figure S5E). In addition, GW501516 did not increase hypoxia-induced PPARδ upregulation (Figure S5F). Taken together, these results suggested that PPARδ enhanced HIF1α target gene expression most likely relied on PPARδ protein upregulation but not ligand driving activation.

Hypoxia also upregulated PPARδ in BMECs at both protein and mRNA level (Figure 5B-C, Figure 6F), which was attenuated at protein level by *Hif1a* siRNA in BMECs (Figure 6F) and also at mRNA level in Hela cells (Figure S4K), indicating that HIF1α might be able to regulate PPARδ transcription. In additional, hypoxia also induced more PPARδ translocation to the nuclei as shown by immunofluorescence in BMECs (Figure 6G), implying that under hypoxia, not only PPARδ expression is increased, but it is also more accessible to interact with HIF1α. Altogether, our results indicated that hypoxia upregulates PPARδ expression by HIF1α and PPARδ reciprocally stabilizes HIF1α protein which could be responsible for post-ischemic vascular repair.

### Expression of stable HIF1α improves the delayed vascular repair due to loss of endothelial PPARδ

**Figure 7:**
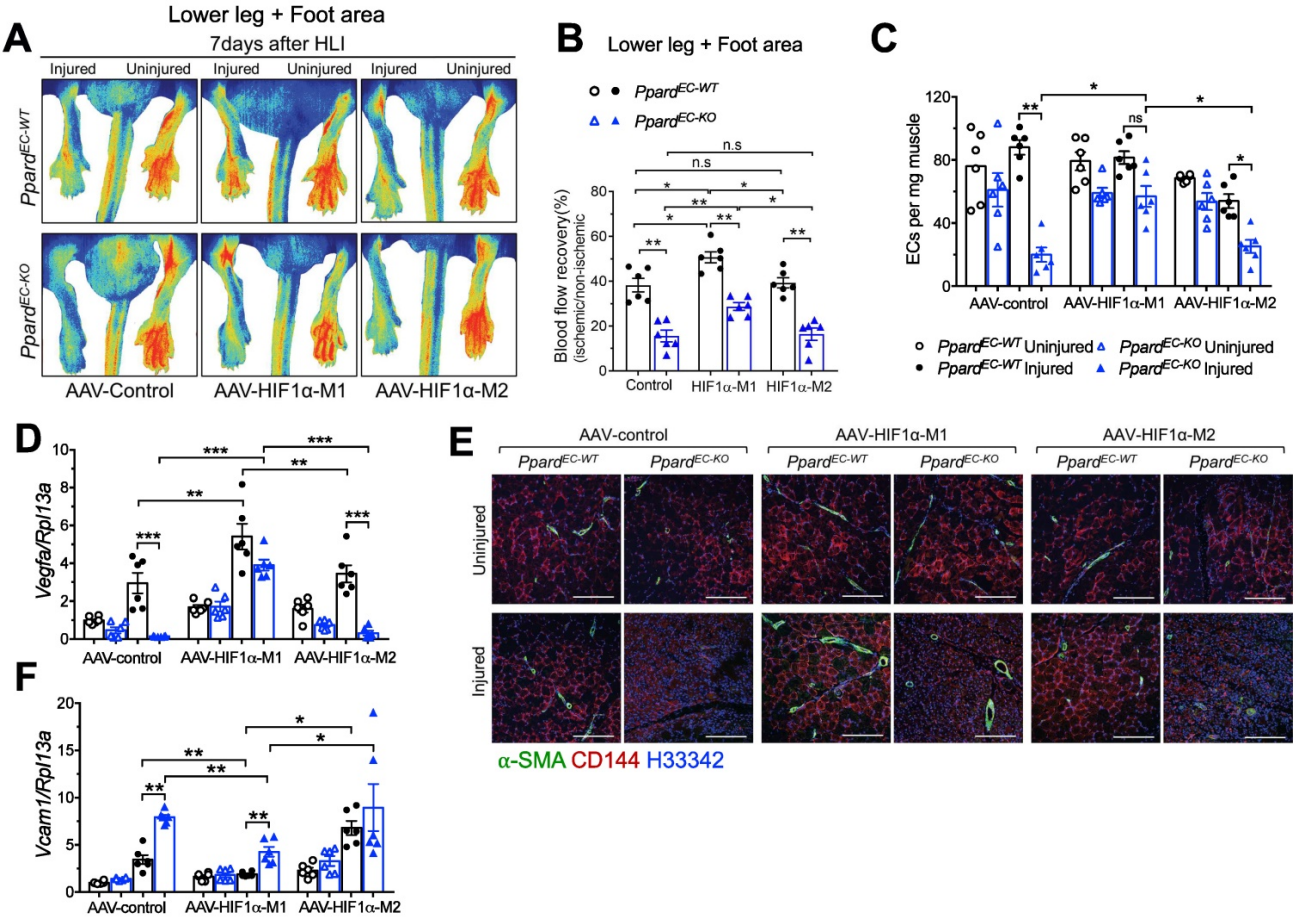
** Expression of stable HIF1α in endothelial cells improves vascular repair. A** (representative images) and **B** (summarized analysis) showing vasculature imaging in mouse foot area recorded at day 7 after HLI (n = 6, each group). **C**, flow cytometric analysis of CD45^-^CD144^+^ECs at day 7 after HLI (n = 6, each group). **D&F**, qPCR analysis for Vegfa (**D**) and Vcam1 (**F**) mRNA expression in muscles collected 7 days after HLI (n = 6, each group). **E**, Representative immunofluorescence of α-SMA co-stained with CD144 in muscle at day 7 after HLI (n = 6, each group). Scale bar: 200 μm. Results are means ± SEM. * p < 0.05, ** p < 0.01, between groups by one-way ANOVA and multiple comparison test.

Because in vitro experiments indicated a strong interaction between PPARδ and HIF1α, we further studied whether stable HIF1α would rescue the delayed vascular repair induced by loss of endothelial PPARδ. To do it, we used AAV to overexpress either the stable and active M1-HIF1α which has P402A/P564A mutation allowing HIF1α to maintain stabilization by preventing its hydroxylation and binding to E3 ubiquitin ligase 36, or the negative control M2-HIF1α (HIF1α-(ΔODD)/R27G), in which the ODD domain was removed. This ODD modification makes HIF1α stable at normoxia but R27G mutation further abolishes the DNA binding ability, which makes the M2-HIF1α stable but lacking transcriptional activity 25. AAV to overexpress M1 or M2 selectively in ECs driven by *Icam2* promoter was injected one week before HLI. Expression of HIF1α from both M1 and M2 could be detected in CD144^+^ ECs (Figure S6A). Expression of HIF1α target genes *Pdk1*, *Adm*, and *Glut1* after HLI was increased more in the muscles from M1 than M2 (Figure S6B-D). Vascular perfusion was enhanced in *Ppard*^EC-KO^ by HIF1α-M1, although the perfusion in *Ppard*^EC-KO^ with HIF1α-M1 was still worse than *Ppard*^EC-WT^, suggesting both HIF1α -dependent and -independent effects regulated by PPARδ (Figure 7A-B). Meanwhile, the effect of HIF1α-M2 was similar to AAV-control. EC numbers as an indicator of post-ischemic angiogenesis were quantified by flow cytometric analysis which showed that HIF1α-M1 but not M2 increased EC number in *Ppard*^EC-KO^ mice at 7 days post-HLI (Figure 7C). Consistently, Vegfa expression was also higher with M1 (Figure 7D). In addition, CD144 and α-SMA upregulations were observed in both *Ppard*^EC-KO^ and *Ppard*^EC-WT^ mice after HIF1α-M1 but not M2 injection (Figure 7E). Importantly, CD144 expression in HIF1α-M1, but not control or M2, was less discontinuous (Figure 7E), indicating better repair of a functional endothelium induced by HIF1α-M1. Likewise, upregulation of Vcam1 was attenuated by HIF1α-M1, whereas M2 remained similar as control (Figure 7F). These results suggested that restoring HIF1α expression and activity improved the vascular repair impaired by endothelial *Ppard* deletion, while some effects of PPARδ might be HIF1α-independent.

## Discussion

In this study, we investigated endothelial selective loss of PPARδ expression in ischemic injury. We found that PPARδ orchestrates many functional aspects of ECs including angiogenesis, vascular reactivity, vascular barrier function, and inflammatory responses, associated with HIF1α signaling. We also found that hypoxia upregulates PPARδ, which interacts and stabilizes HIF1α, during which the two transcription factors enhance the expression and transactivation of each other.

Although several previous studies showed the effect of PPARδ in ECs and other vascular cells, many were based on the effect of ligands, with little known about how PPARδ responds and changes to vascular injury. The effect of PPARδ on angiogenesis was mostly only observed in isolated ECs using pharmacological ligands. PPARδ ligands including L-165041, GW501516, and prostacyclin 37, enhance angiogenesis and prevent apoptosis in human EPCs 18, 21. These human EPCs, when injected into mice, showed impaired angiogenesis with silencing of PPARδ 21. PPARδ ligands also enhance angiogenesis by regulating GTPCHI and BH_4_ related to eNOS activity 19, as well as upregulation of angiogenic factors like VEGFs 37. However, the influence of *in vivo* loss of PPARδ on angiogenesis or other functions of EC, in addition to vascular tone, remains unclear. The involvement of endogenous PPARδ has only been shown recently using global *Ppard* knockout mice which suggested a reduced retinal angiogenesis and vessel remodeling only at steady state 38. In the present study, we found that ischemia-induced angiogenesis and possibly vasculogenesis was impaired in the *Ppard*^EC-KO^ mice, suggested by a delayed appearance of capillary ECs and arterioles, accompanied by the failure to upregulate many angiogenic factors including the VEGF signaling.

In addition to regeneration of vasculature, restoration of endothelial barrier function is also important for recovery of microvessel function in PAD. Previous studies on PPARδ mostly focused on other vasculatures excluding the muscle capillaries. GW0742 help to reduce blood brain barrier leakage after brain injury 39. However, opposite effect was observed in retinal ECs, using *PPARD* siRNA and inhibitor to reduce VEGF-induced hyperpermeability 40. We speculate that the opposite effect might be due to the different responses of PPARδ under different oxygen tension, and therefore, might be influenced by HIF1α. Furthermore, delayed recovery of barrier function from both the existing and newly regenerated ECs in the *Ppard*^EC-KO^ mice might lead to more persistent endothelial activation and vascular inflammation after HLI. Although PPARδ ligands have been known for its potent anti-inflammatory effects, we showed here a previously unrecognized contribution of endogenous PPARδ against vascular inflammation in response to ischemic injury. All these results suggested an important role of PPARδ in restoring vascular homeostasis after ischemic injury. The vascular phenotype of *Ppard*^EC-KO^ mice was also under-explored. Using a different strain of endothelial Cre, the Tie2-Cre to generate endothelial selective Ppard knockout mice (*Ppard*^floxed^;*Tie*^Cre/+^ mice), another group showed a small but significant impairment of endothelium-dependent relaxation in the aorta in response to ACh under unstimulated condition due to increased H_2_O_2_ production which decreased NO availability 41. Such differences might be due to sensitivity of ligand and to NO in femoral arteries different from aorta, and also possibly due to strain differences.

To provide a more mechanistic role of how endothelial PPARδ regulates vascular homeostasis, we further studied the role of HIF1α. The present results suggested that endogenous PPARδ could be activated in response to hypoxic stress. Notably, PPARδ regulates HIF1α protein by reducing HIF1α degradation in ECs under hypoxia. Such effect was likely due to the interaction between the LBD domain of PPARδ and the ODD domain of HIF1α. To further confirm the regulation of HIF1α by PPARδ *in vivo*, we used AAV to overexpress stable HIF1α which was able to ameliorate the delayed vascular repair due to loss of PPARδ. Interestingly, this mechanism of ligand-independent regulation of HIF1α by PPARδ from our study is different from a previous study which showed overlapping of transcriptome regulation in PPARδ agonist -treated and hypoxia-treated human ECs 42. Future study to assess the effect of PPARδ ligand on angiogenesis in the *Ppard*^EC-KO^ mice, which might strengthen the current finding. It is also unclear whether and which endogenous ligand(s) is playing a major role in the activation of PPARδ after HLI. Nevertheless, these results suggested a ligand-independent role of PPARδ to respond to hypoxia and to facilitate the restoration of vascular homeostasis through enhancing HIF1α function.

The role of HIF1α in post-ischemic vascular responses has been well established by studies using either gain- or loss-of-function 43, 44. Apart from angiogenesis, HIF1α also regulates other functions of ECs such as stimulating proliferation, inhibiting microvascular leakage and enhances vascular repair 45. Several proteins such as NQO1, Runx2 46 and CBX4 47, etc., have been identified to interact with the ODD domain of HIF1α, and as a result, enhances HIF1α stability and HIF1α-mediated angiogenesis. Here we showed a new role of PPARδ to stabilize HIF1α in EC, which acts through a similar mechanism through binding to the ODD domain. Although ligand activation of PPARδ reduces the association of HIF1α, HIF1α is unlikely to be a co-repressor acting like Cry1/2 28, because unlike Cry1/2, silencing of HIF1α did not increase PPARδ target gene expression. Quite the contrary, HIF1α upregulated PPARδ, suggesting that HIF1α acts as co-activator of PPARδ, whereas ligand activation of PPARδ does not facilitate HIF1α stabilization and transactivation.

In conclusion, we showed a central role of endothelial PPARδ in vascular homeostasis and post-ischemic vascular repair by regulating gene regulatory network involved in angiogenesis, endothelial barrier function, and vascular inflammation. PPARδ is induced by hypoxia in endothelial cells and it reciprocally enhances HIF1α stability and transactivation. These results also provide new information about a ligand independent activation of PPARδ in vasculature through its interaction with HIF1α, in response to hypoxic stress.

## Supplementary Material

Supplementary figures and tables.

## Abbreviations

AAV: adeno-associated virus
BMEC: brain microvascular endothelial cell
CHX: cycloheximide
DBD: DNA-binding domain
EC: Endothelial cell
FITC: Fluorescein isothiocyanate
GA: gastronemius
HIF1α: hypoxia-inducible factor 1-alpha
HLI: hindlimb ischemia
LBD: ligand-binding domain
ODD: oxygen dependent degradation domain
PAD: peripheral artery disease
PPARδ: peroxisome proliferator activated Receptor delta
