# Supplementary material for: Endothelial PPARδ facilitates the post-ischemic vascular repair through interaction with HIF1α
Source: Theranostics. 2022 Jan 24;12(4):1855–69. doi: 10.7150/thno.69017 (PMC8825601; doi:10.7150/thno.69017)
Supplement: Supplementary file 1 — Supplementary figures and tables. [file thnov12p1855s1.pdf]

Supplementary Figures

Supplementary Figure 1

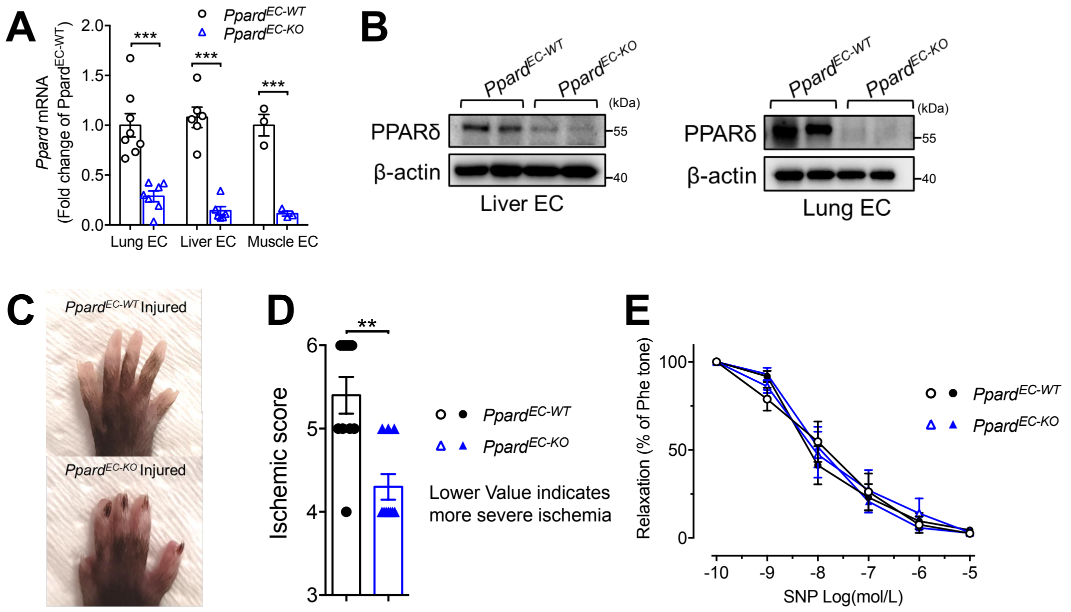

Figure S1. PPARδ expression Endothelial cells were isolated from lung, liver and skeletal muscle measured at mRNA level by qRT -PCR (**A**) (n = 8 for lung group, n = 6 for liver group, n = 3 for skeletal muscle group) and at protein level by Western blotting (**B**). **C**, Representative pictures of the foot color and nails at days 7 after HLI. **D**, Ischemic score as described (n = 10, each group). **E**, Concentration-response curves to SNP in femoral arteries at days 28 after HLI (n = 6, each group). Results are means ± SEM. Student's t test was used for comparison between two samples, and one-way ANOVA and multiple comparison test was used for more than two samples. * $p < 0.05$, ** $p < 0.01$, *** $p < 0.001$ between groups.

Supplementary Figure 2

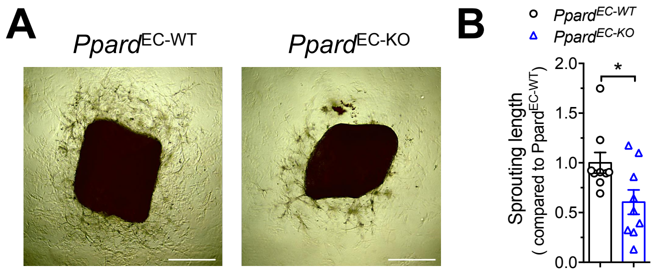

Figure S2. **A** (representative images) and **B** (summarized analysis) of sprouting length from mouse aortic ring in *Ppard*$^{EC\text{-}KO}$ mice compared to *Ppard*$^{EC\text{-}WT}$ (n = 9, each group). Scale bar: 200 µm. Student's t test was used for comparison between two samples, and one-way ANOVA and multiple comparison test was used for more than two samples. * $p < 0.05$, ** $p < 0.01$, *** $p < 0.001$ between groups.

Supplementary Figure 3

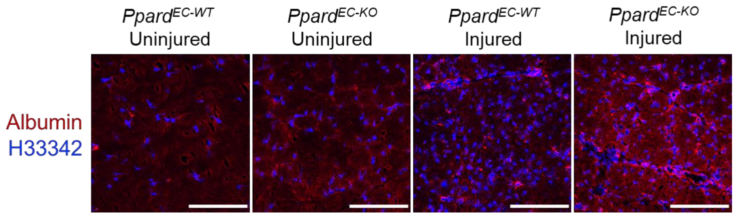

Figure S3. Representative images of immunostaining of albumin (red) in frozen sections of GA muscle 14 days after HLI (n = 6, each group). Scale bar: 200 µm.

Supplementary Figure 4

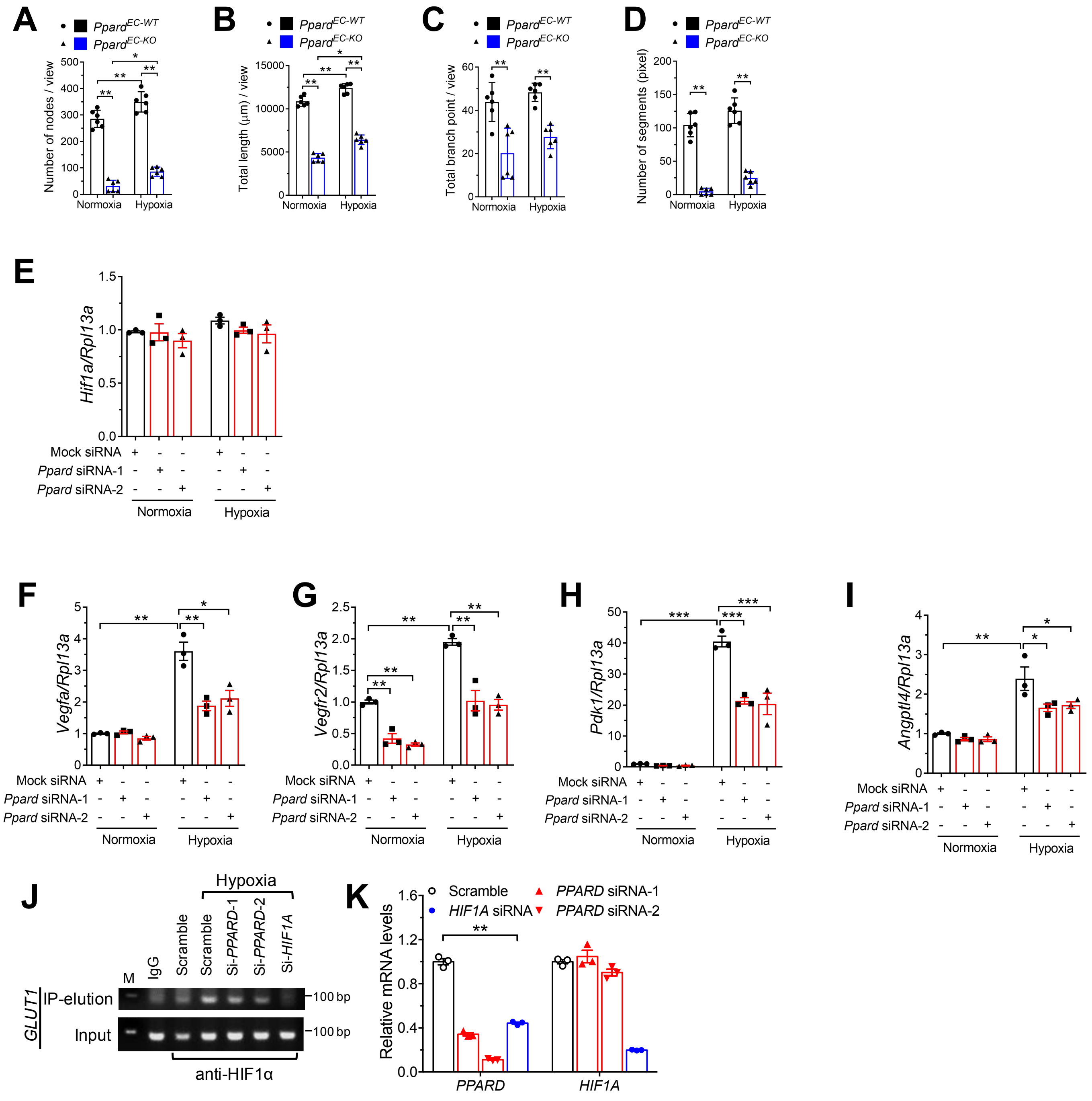

Figure S4. A-D, summarized analysis of tube formation in **Figure. 5A** analyzed using the Angiogenesis Analyzer of Image J (n = 6, each group). **E-I**, qPCR analysis of *Hif1a*, *Vegfa, Vegfr2, Pdk1, and Angptl4* mRNA levels in mBMECs under hypoxia for 12 h after transfection with siRNA, from 3 biological replicates. **J**, Representative gel showing ChIP of anti-HIF1α immunoprecipitates from Hela cells after *HIF1A* or *PPARD* silencing, followed by hypoxia for 12 h to show HIF1α enrichment on the *GLUT1* promoter. **K**, mRNA expression of HIF1α or PPARδ under hypoxia for 12 h. in Hela cells. Results are means ± SEM. * $p < 0.05$, ** $p < 0.01$ between groups by one-way ANOVA and multiple comparison test.

Supplementary Figure 5

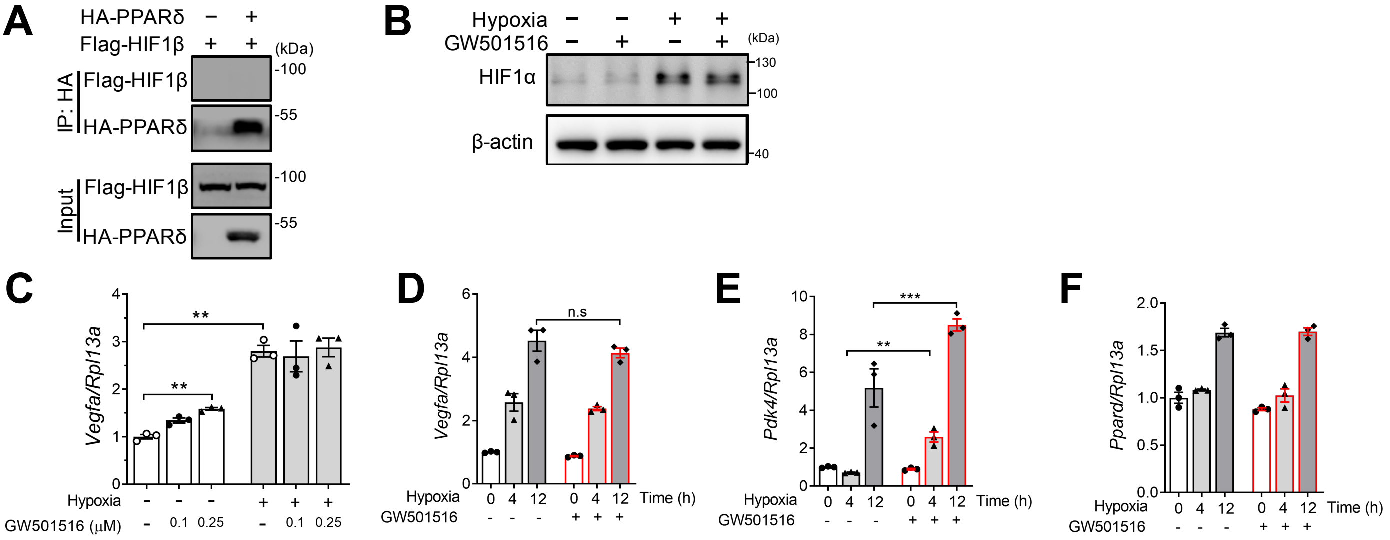

Figure S5. A, Immunoblots showing the anti-HA immunoprecipitates in HEK293T cells with indicated plasmids transfected, indicating the no interaction of PPARδ and HIF1β in HEK293T cells. **B**, Immunoblots showing HIF1α under indicated conditions in BMECs (Hypoxia: 12 hours) with or without GW501516 (100 nmol/L). Results are representative data with at least three replicates. **C-F**, qPCR analysis of *Vegfa*, *Pdk4*, and *Ppard* in mBMECs under hypoxia for 12 h after treated with GW501516 at the indicated concentration and duration. Results are biological replicates expressed in means ± SEM. * $p < 0.05$, ** $p < 0.01$ between groups by one-way ANOVA and multiple comparison test.

Supplementary Figure 6

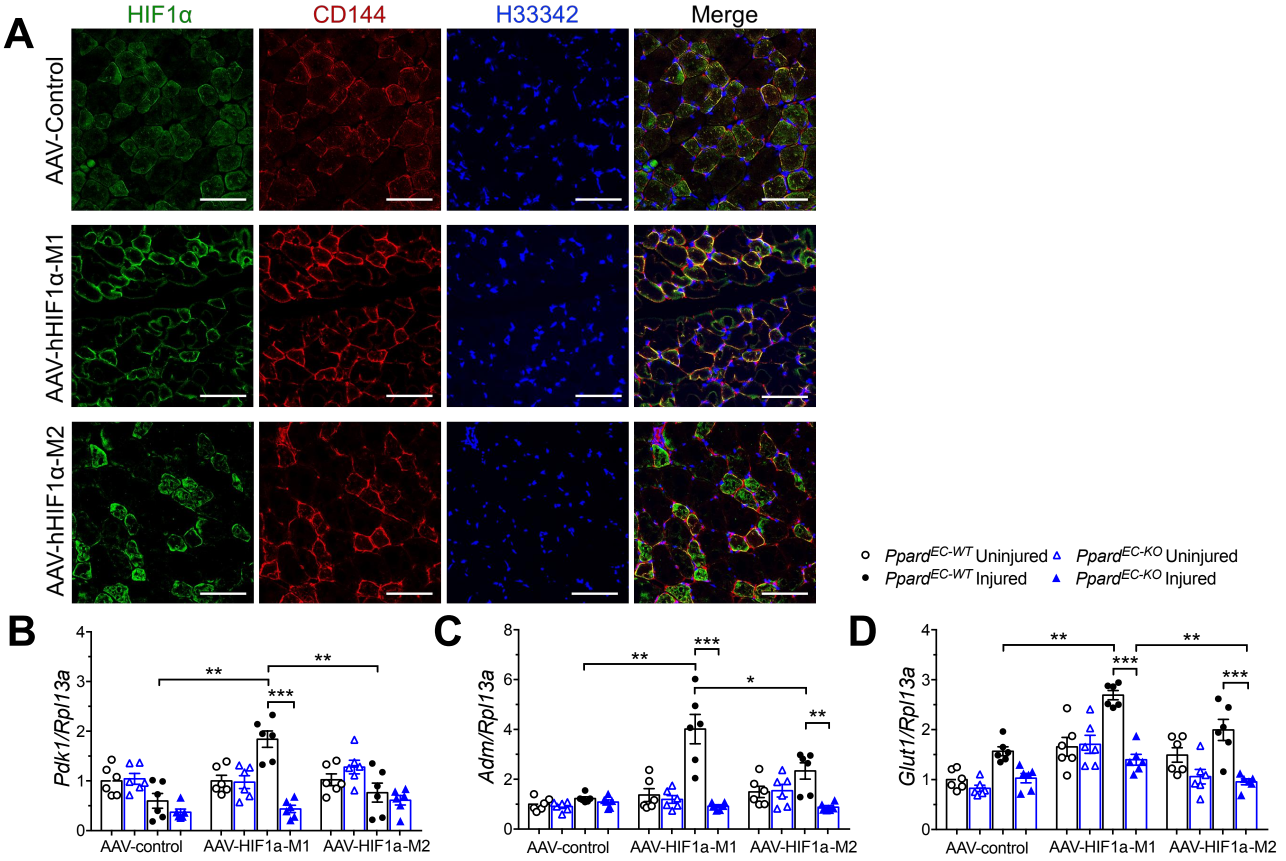

Figure S6. A, Representative images of immunofluorescence of HIF1α (green) expression to co-localized with CD144 (red) in muscle at day 7 after HLI (n = 3, each group). Scale bar: 200 μm. **B-D**, qPCR analysis for HIF1α downstream gene mRNA expression in muscles collected 7 days after HLI(n = 6, each group). Results are means ± SEM. * $p < 0.05$, ** $p < 0.01$, between groups by one-way ANOVA and multiple comparison test.

Table S1: List of primers

Genes	Primer sequence (5' to 3')
Mouse *18sRNA*	GCAATTATTCCCCATGAACG<br>GGCCTCACTAAACCATCCAA
Mouse *Gapdh*	ATGGTGAAGGTCGGTGTGAA<br>GAGGTCAATGAAGGGGTCGT
Mouse *Pax3*	GCGTCTCTAAGATCCTGTGCAG<br>GATTTCCCAGCTAAACATGCCCG
Mouse *Pax7*	GTTCGGGAAGAAAGAGGACGAC<br>GGTTCTGATTCCACATCTGAGCC
Mouse *Myf5*	GGTGGAGAACTATTACAGCCTGC<br>ACAGTAGATGCTGTCAAAGCTGC
Mouse *MyoD*	GCACTACAGTGGCGACTCAGAT<br>TAGTAGGCGGTGTCGTAGCCAT
Mouse *Myogenin*	CCATCCAGTACATTGAGCGCCT<br>CTGTGGGAGTTGCATTCACTGG
Mouse *α-SMA* (*Acta2*)	TGCTGACAGAGGCACCACTGAA<br>CAGTTGTACGTCCAGAGGCATAG
Mouse *CD31*(*Pecam1*)	AGTCAGAGTCTTCCTTGCCC<br>CTCCACGGGTTTCTGTTTGG
Mouse *Cxcl12*	GCCAACGTCAAGCATCTGAA<br>TTCGGGTCAATGCACACTTG
Mouse *Aqp1*	CTTGCCATTGGCTTGTCTGTGG<br>CCAGTGGTTTGAGAAGTTGCGG
Mouse *Klf2*	CACCTAAAGGCGCATCTGCGTA<br>GTGACCTGTGTGCTTTCGGTAG
Mouse *Fabp4*	AAGGTGAAGAGCATCATAACCCT<br>TCACGCCTTTCATAACACATTCC
Mouse *Eglf7*	CTGAGCATGTCTACAGACCCAG<br>TCTGTGTCCGTCGCAAGTGGTG
Mouse *Afdn*	TGCCAGCCTTTCTGGATGATCC<br>CTGGATGGTCAAGGCAGCATTG
Mouse *Jam-2*	CAGACTGGAGTGGAAGAAGGTG<br>GCTGACTTCACAGCGATACTCTC
Mouse *Jam-3*	GCATTGCTTCCAATGACGCAGG<br>GATGAAGCAGCCTCGTCTGTAC
Mouse *Nectin-2*	GCCATACTGACCTGTGATGTACG<br>TCCACAGAGTGGACAAGCAGCT
Mouse *Gja5*	GTGCCAAACCAGGAGCAGATTC<br>CGCCGTTTGTCACTATGGTAGC
Mouse *Tie2*	GAACTGAGGACGCTTCCACATTC<br>TCAGAAACGCCAACAGCACGGT
Mouse *Vegfa*	GCTGTAACGATGAAGCCCTG<br>CCTATGTGCTGGCTTTGGTG
Mouse *Apelin*	ATGAATCTGAGGCTCTGCGT<br>GTCCTCGAAGTTCTGGGCTT
Mouse *Vegfr2*	TTTGGCAAATACAACCCTTCAGA<br>GCAGAAGATACTGTCACCACC
Mouse *Angiop-1*	AGGCTTGGTTTCTCGTCAGA<br>TGTCCATGAGCTCCAGTTGT
Mouse *IL-8*	GGTGATATTCGAGACCATTTACTG<br>GCCAACAGTAGCCTTCACCCAT
Mouse *Fgf2*	AAGCGGCTCTACTGCAAGAACG

	CCTTGATAGACACAACTCCTCTC
Mouse *Hgf*	GCAGTACCCTCACAAGCATG<br>ACTCGGATGTTTGGGTCAGT
Mouse *Cldn5*	GCAAGGTGTATGAATCTGTGCT<br>GTCAAGGTAACAAAGAGTGCCA
Mouse *Zo-1*	GTTGGTACGGTGCCCTGAAAGA<br>GCTGACAGGTAGGACAGACGAT
Mouse *Occludin-1*	TGGCAAGCGATCATACCCAGAG<br>CTGCCTGAAGTCATCCACACTC
Mouse *Nectin-1*	AGCGGACAGATGTGAAGCTCAC<br>TTCCTGCCAGGCTGTAGGTGAT
Mouse *Icam1*	AAACCAGACCCTGGAACTGCAC<br>GCCTGGCATTTCAGAGTCTGCT
Mouse *Vcam1*	ACAGACAGTCCCCTCAATGG<br>TCCTCAAAACCCACAGAGCT
Mouse *E-selectin*	AGTTGTGAGTTCTCCTGCGA<br>CACTCCATGACGCCATTCTG
Mouse *Ccl2*	CATCCACGTGTTGGCTCA<br>GATCATCTTGCTGGTGAATGAGT
Mouse *Ccr2*	ACCAGAAGAGGGCATTGGAT<br>GCCGTGGATGAACTGAGGTA
Mouse *Cx3cl1*	GTGCGACAAGATGACCTCAC<br>GCGTCTTGGACCCATTTCTC
Mouse *IL-1β*	GAAATGCCACCTTTTGACAGTG<br>TGGATGCTCTCATCAGGACAG
Mouse *IL-6*	TCTATACCACTTCACAAGTCGGA<br>GAATTGCCATTGCACAACTCTTT
Mouse *IFNγ* (*IFNG*)	CAGCAACAGCAAGGCGAAAAAGG<br>TTTCCGCTTCCTGAGGCTGGAT
Mouse *Hif1α*	ACTTTTGGGCCGCTCAATTT<br>ACTTTTGGGCCGCTCAATTT
Mouse *Glut1*	GCTTCTCCAACTGGACCTCAAAC<br>ACGAGGAGCACCGTGAAGATGA
Mouse *Angptl4*	CTGGACAGTGATTCAGAGACGC<br>GATGCTGTGCATCTTTTCCAGGC
Mouse *Rpl13a*	AGTATCTGGCCTTTCTCCGG<br>CCGAACAACCTTGAGAGCAG
Mouse *Pdk1*	CTGAGCATGTCTACAGACCCAG<br>TCTGTGTCCGTCGCAAGTGGTG
Mouse *Ppard*	AGGAGAAAGAGGAAGTGGCC<br>GGGAGGAATTCTGGGAGAGG
Mouse *Adm*	GCCAGATACTCCTTCGCAGTTC<br>AGGAACTGTCGTCTCATCAGCG
Human *Hif1α*	GCCACATCATCACCATATAGAG<br>GACTCAAAGCGACAGATAACAC
Human *Ppard*	GGCTTCCACTACGGTGTTCATG<br>CTGGCACTTGTTGCGGTTCTTC
Human *Glut1*<br>(For ChIP)	TAGCAACAGCGAGCGTGCCG<br>CCCCGTCGTTTGGTCTCCT
PPARδ-DBD	ATTCATCGATAGATCTGATGGAGCAGCCACAGGAGGAAGC<br>CC<br>ATGCCACCCGGGATCCTTACAGCCCTGCCACCAGCTTCCT<br>CTTC

P*PARδ*-LBD	ATTCATCGATAGATCTGCAGGTGGCCGACCTGAAGGCCTTCTC<br>ATGCCACCCGGGATCCTTAGTACATGTCCTTGTAGATCTCCT

Table S2: List of antibodies

Target antigen	Source	Catalog	Working concentration
Anti-alpha smooth muscle Actin	Abcam	ab32575	1:500
AF647 Rat Anti-Mouse CD144	BD biosciences	562242	1:100
Alexa Fluor® 488 anti-mouse CD31	MEC13.3	Biolegend	1:200
PE-Cy™7 Rat Anti-CD11b	BD biosciences	552850	1:100
Anti-Albumin	Abcam	ab207327	1:500
CD144 (VE-cadherin) Monoclonal Antibody	Invitrogen	14-1441-82	1:500
Brilliant Violet 605™ anti-mouse CD45 Antibody	Biolegend	103140	1:200
BV605 Rat Anti-Mouse CD144	BD Biosciences	748261	1:100
PE/Cy7 anti-mouse F4/80	Biolegend	123114	1:100
Anti-CD68 antibody	Abcam	ab31630	1:250
PerCP/Cy5.5 anti-mouse Ly-6C Antibody	Biolegend	128012	1:100
VEGFR1 antibody	Affbiotech	AF6204	1:250
CD81 antibody-internal	Affbiotech	DF2306	1:250
CD63 antibody-internal	Affbiotech	DF2305	1:250
Claudin 5 antibody-C-terminal	Affbiotech	AF5216	1:250
HIF1A antibody	Affbiotech	BF0593	1:500
LIVE/DEAD™ Fixable Aqua Dead Cell Stain Kit	Invitrogen™	L34966	1:500
Goat anti-Rabbit IgG (H+L) Highly Cross-Adsorbed Secondary Antibody, Alexa Fluor 488	Invitrogen	A-11034	1:500
Anti-PPAR delta antibody (ab23673)	Abcam	ab23673	1:1000
Goat Anti-Rabbit IgG (H + L)-HRP Conjugate	Bio-rad	1706515	1:2000
Goat Anti-Mouse IgG (H + L)-HRP Conjugate	Bio-rad	1706516	1:2000
Brilliant Violet 605™ anti-mouse CD64 (FcγRI) Antibody	Biolegend	139323	1:100
Alexa Fluor® 647 anti-mouse/human Ki-67 Antibody	Biolegend	151206	1:100
PE anti-mouse CD106 Antibody	Biolegend	105713	1:100
mouse anti-beta-actin loading control antibody	Thermofisher	MA5-15739	1:1000
mouse anti-GAPDH loading control antibody	Thermofisher	MA5-15738	1:1000
PE anti-mouse Ly-6G Antibody	Biolegend	127608	1:100
AF488 anti-mouse CD4 antibody	Biolegend	100529	1:100
Recombinant Anti-VCAM1 antibody	Abcam	ab134047	1:250
Purified Rat Anti-Mouse CD16/CD32 (Mouse BD Fc Block™)	BD Pharmingen™	553142	1:1000
ZO-1 Monoclonal Antibody (ZO1-1A12)	Invitrogen	33-9100	1:100

HIF-1α (D1S7W) XP® Rabbit mAb #36169	CST	36169S	1:100
Goat anti-Mouse IgG (H+L) Highly Cross-Adsorbed Secondary Antibody, Alexa Fluor Plus 488	Invitrogen	A32723	1:500
Goat anti-Rat IgG (H+L) Cross-Adsorbed Secondary Antibody, Alexa Fluor 568	Invitrogen	A-11077	1:500
PE/Cy7 anti-mouse CD133 Antibody	Biolegend	315-2C11	1:100
Mouse VEGFR2/KDR/Flk-1 Antibody	R&D	Af644	1:100
Anti-CD31 antibody	Abcam	ab28364	1:500
Fluorescein labeled Griffonia (Bandeiraea) Simplicifolia Lectin I (GSL I, BSL I)	Vectorlabs	FL-1101-5	
